# Multimodal Imaging of Torpedo Maculopathy With Fluorescence Adaptive Optics Imaging of Individual Retinal Pigmented Epithelial Cells

**DOI:** 10.3389/fmed.2021.769308

**Published:** 2021-12-09

**Authors:** Kari V. Vienola, Kunal K. Dansingani, Andrew W. Eller, Joseph N. Martel, Valerie C. Snyder, Ethan A. Rossi

**Affiliations:** ^1^Department of Ophthalmology, University of Pittsburgh, Pittsburgh, PA, United States; ^2^Department of Bioengineering, University of Pittsburgh Swanson School of Engineering, Pittsburgh, PA, United States; ^3^McGowan Institute for Regenerative Medicine, University of Pittsburgh, Pittsburgh, PA, United States

**Keywords:** torpedo maculopathy, retinal pigment epithelium, autofluorescence, adaptive optic systems, scanning laser ophthalmoscopy, fluorescence imaging

## Abstract

Torpedo maculopathy (TM) is a rare congenital defect of the retinal pigment epithelium (RPE). The RPE is often evaluated clinically using fundus autofluorescence (AF), a technique that visualizes RPE structure at the tissue level from the intrinsic AF of RPE fluorophores. TM lesions typically emit little or no AF, but this macroscopic assessment is unable to resolve the RPE cells, leaving the organization of the RPE cell mosaic in TM unknown. We used fluorescence adaptive optics scanning laser ophthalmoscopy (AOSLO) to show here for the first time the microscopic cellular-level structural alterations to the RPE cell mosaic in TM that underlie the tissue-level changes seen in conventional clinical imaging. We evaluated two patients with TM using conventional clinical imaging techniques and adaptive optics (AO) infrared autofluorescence (IRAF) in AOSLO. Confocal AOSLO revealed relatively normal cones outside the TM lesion but altered cone appearance within it and along its margins in both patients. We quantified cone topography and RPE cell morphometry from the fovea to the margin of the lesion in case 1 and found cone density to be within the normal range across the locations imaged. However, RPE morphometric analysis revealed disrupted RPE cells outside the margin of the lesion; the mean RPE cell area was greater than two standard deviations above the normative range up to approximately 1.5 mm from the lesion margin. Similar morphometric changes were seen to individual RPE cells in case 2. Multi-modal imaging with AOSLO reveals that RPE cells are abnormal in TM well beyond the margins of the characteristic TM lesion boundary defined with conventional clinical imaging. Since the TM fovea appears to be fully formed, with normal cone packing, it is possible that the congenital RPE defect in TM occurs relatively late in retinal development. This work demonstrates how cellular level imaging of the RPE can provide new insight into RPE pathologies, particularly for rare conditions such as TM.

## Introduction

Torpedo maculopathy (TM) is characterized by the presence of a well-circumscribed lesion of the macula featuring elements of hyperpigmentation and atrophy at the level of the retinal pigment epithelium (RPE) and named for its typical shape and orientation with respect to the fovea (1, 2). TM appears to be congenital, non-progressive, and uncommon. Prevalence has been estimated to be 2 per 100,000 (3) with fewer than 100 cases reported as of 2019 (4). Early conjectures about the most appropriate classification included a subclass of congenital nevus of the RPE, or a form of congenital hypertrophy of the RPE (5, 6), but these descriptions pre-date optical coherence tomography (OCT).

In TM, OCT sometimes reveals neurosensory retinal layers detached from the RPE, forming a cavity (7, 8). Based on this, Wong et al. classified TM into Types I and II, the latter including neurosensory detachment with subretinal cavitation (9). Often the OCT B-scans show increased light penetration to the choroid with minimal fundus autofluorescence (FAF) signal originating from the lesion (2, 10). Functional testing with visual fields (11) and microperimetry (9) has shown reduced sensitivity in the lesion. Collective responses from electroretinogram (ERG) exams performed on patients with TM have not shown any abnormalities (12). However, multi-focal ERG has shown amplitude reduction with potential latency between pathological and healthy retinal areas of the same eye (12, 13).

In recent years, OCT angiography (OCTA) has been used to evaluate flow profiles in the choriocapillaris and/or choroid in the lesions. Most of these studies have reported decreased flow in the choriocapillaris with vascular alterations at the site of the lesion (11, 14–16). Giannakaki-Zimmermann et al. (17) concluded that attenuation of OCTA signal in choriocapillaris occurs with Type I as well, when there is no subretinal cavitation on structural OCT. Although one publication reported an increase in choroidal vascular density, no explanation was offered as to how this was measured and closer inspection of the key figure in the paper shows reduced choriocapillaris flow density in the lesion, compared with areas outside the lesion (18).

Adaptive optics ophthalmoscopy (AOO) permits cellular level imaging of the retina (19, 20). Recently, Hugo et al. used a commercially available flood-illumination adaptive optics (FIAO) fundus camera to evaluate TM patients and showed decreased cone density in the lesion compared to the healthy retina, suggestive of cone loss (21). Similar findings were reported by Lambart et al. (22) however, they hypothesized that cones were axially displaced rather than lost. Here, we present two new patients with TM who were evaluated using standard clinical imaging tools and AOO, including a commercial FIAO retinal camera and a custom fluorescence adaptive optics scanning laser ophthalmoscope (AOSLO). Compared to FIAO, AOSLO can achieve higher resolution for “reflectance” (i.e., backscattered) light imaging due to its ability to reject out-of-focus light (confocality). Our AOSLO is also equipped with a near-infrared autofluorescence detection channel that we used here to define the morphological alterations to the RPE cell mosaic associated with TM. To our knowledge, this is the first time that RPE morphology has been examined and quantified *in vivo* in TM patients using any modality.

Given the well-circumscribed nature of TM, our hypothesis was that the transition from abnormal to normal cellular mosaics would be relatively abrupt, with abnormalities confined to the immediate vicinity of the lesion. Further, due to the non-progressive clinical course, we hypothesized that the demarcation between abnormal and normal photoreceptor and RPE mosaics would be approximately aligned, without one appearing to lead or lag the other in terms of distance from the lesion border.

## Methods

Clinical imaging included color fundus photography (Topcon/Canon), SLO/OCT (Spectralis, Heidelberg, Germany), and a flood-illumination adaptive optics camera (rtx1-e, Imagine Eyes, France). Detailed OCT imaging parameters are presented in Table 1. Microperimetry (MAIA, CenterVue S.p.A, Padova, Italy) was performed using both the macular test and with custom case-specific test patterns that we used to evaluate light sensitivity across the lesions. Before the testing started, optic nerve head was used to calibrate the measurement and for normal control data we used the normative database of the manufacturer. For patient 1, OCTA was also performed (Optovue, Fremont, CA, USA) using an 8 × 8 mm scan area.

**Table 1:** Optical coherence tomography imaging parameters.

**OCT parameters**	**Patient 1**	**Patient 2**	
	**2019**	**2017**	**2019**
Size X	1,536 pixels (9.8 mm)	768 pixels (9.1 mm)	1,024 pixels (6.1 mm)
Size Z	496 pixels (1.9 mm)	496 pixels (1.9 mm)	496 pixels (1.9 mm)
Scaling X	6.41 μm/pixel	11.79 μm/pixel	6.00 μm/pixel
Scaling Z	3.87 μm/pixel	3.87 μm/pixel	3.87 μm/pixel
ART Mode	ON (16 averaged)	ON (9 averaged)	ON (15 averaged)
Quality	38 dB	42 dB	26 dB
EDI Mode	ON	OFF	ON
Number Of B-Scans	49	61	49
Pattern Size	30° × 10° (9.8 × 3.3 mm)	30° × 25° (9.1 × 7.5 mm)	20° × 20° (6.1 × 6.1 mm)
Distance between B-Scans	68 μm	126 μm	128 μm

Adaptive optics scanning laser ophthalmoscopy imaging was carried out using a system that has been described in detail (23). Briefly, two imaging channels were used for simultaneous confocal reflectance and AO-IRAF imaging. A 795 nm super-luminescent diode was used for simultaneous confocal illumination and AO-IRAF excitation, with AF emission detected between 814 and 850 nm using a double-stacked bandpass filter (FF01-832/37, Semrock, USA). To ensure that no excitation light leaked into the AF detection channel, double-stacked filters were used in the 795 nm illumination path (ET775/50x, Chroma, Bellows Falls VT, USA) to block any spontaneous emission in longer bands. The adaptive optics subsystem used a 909 nm laser diode beacon to detect the ocular aberrations and correct them in closed-loop mode with a deformable mirror. Imaging data were acquired at 30 Hz across a 1.5° × 1.5° field of view for a duration of 60–70 s.

The confocal images were used to co-register the weak signals in the AO-IRAF images using custom strip-based image registration software (24), permitting averaging to increase the signal-to-noise ratio in the AO-IRAF images. For the cell quantification from the AO-IRAF images, a semi-automated algorithm was used (25).

Patients were recruited through the clinics of the UPMC Eye Center. Written informed consent was obtained from both subjects following an explanation of experimental procedures and risks both verbally and in writing. All experiments were approved by the University of Pittsburgh Institutional Review Board and adhered to the tenets of the Declaration of Helsinki. To ensure safe imaging, all light levels were kept below the ANSI laser safety limits (26) and were calculated in accordance with best practices for multi-wavelength ophthalmic imaging (27).

## Results

Patient 1, a woman in her early 30s was found to have a pigmented lesion in her right eye during pre-screening for laser refractive surgery that was diagnosed later as TM. Best corrected visual acuity (BCVA) was 20/15 in each eye. The patient had no history of retinal laser nor injections prior to our imaging. Patient 2 was a woman in her early 20s diagnosed with TM. Her BCVA was 20/20 in each eye. The left eye had received a single intravitreal bevacizumab injection 2 years earlier for neovascularization within the lesion. Her angiography images have been published previously (28).

**Figure 1:**
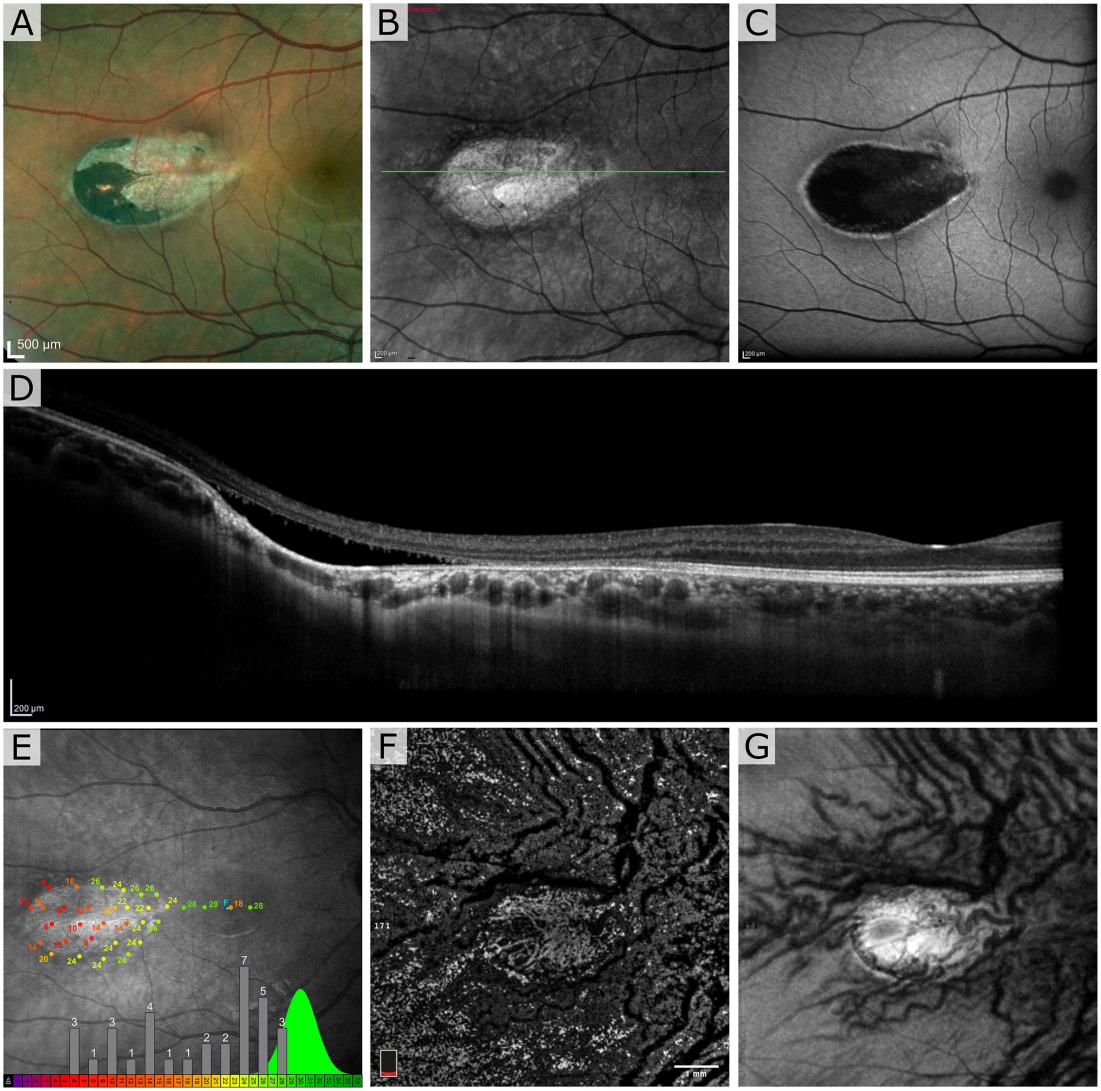
Patient 1 imaged with multimodal clinical imaging. **(A)** Fundus images show a distinct torpedo-shaped lesion at the temporal macular region with the red box indicating the areas for the scanning laser ophthalmoscopy (SLO) and blue autofluorescence (BAF) images. **(B,C)** SLO reflectance and BAF image of the lesion showing the lesion very reflective in the SLO image and exhibiting marked hypo-AF signal at the lesion with a rim of hyper-AF. The green line in **(B)** indicates the B-scan location. **(D)** Optical coherence tomography (OCT) B-scan through the lesion. **(E)** Microperimetry test showing normal sensitivity in the fovea and severely decreased sensitivity at lesion with overall sensitivity well outside the normal distribution. The green distribution represents reference data from normal subjects. OCT *en face* angiogram **(F)** projection and corresponding structural image **(G)** taken with the OptoVue using a slab thickness of 70 μm about 150 μm below the retinal pigment epithelium (RPE).

**Figure 2:**
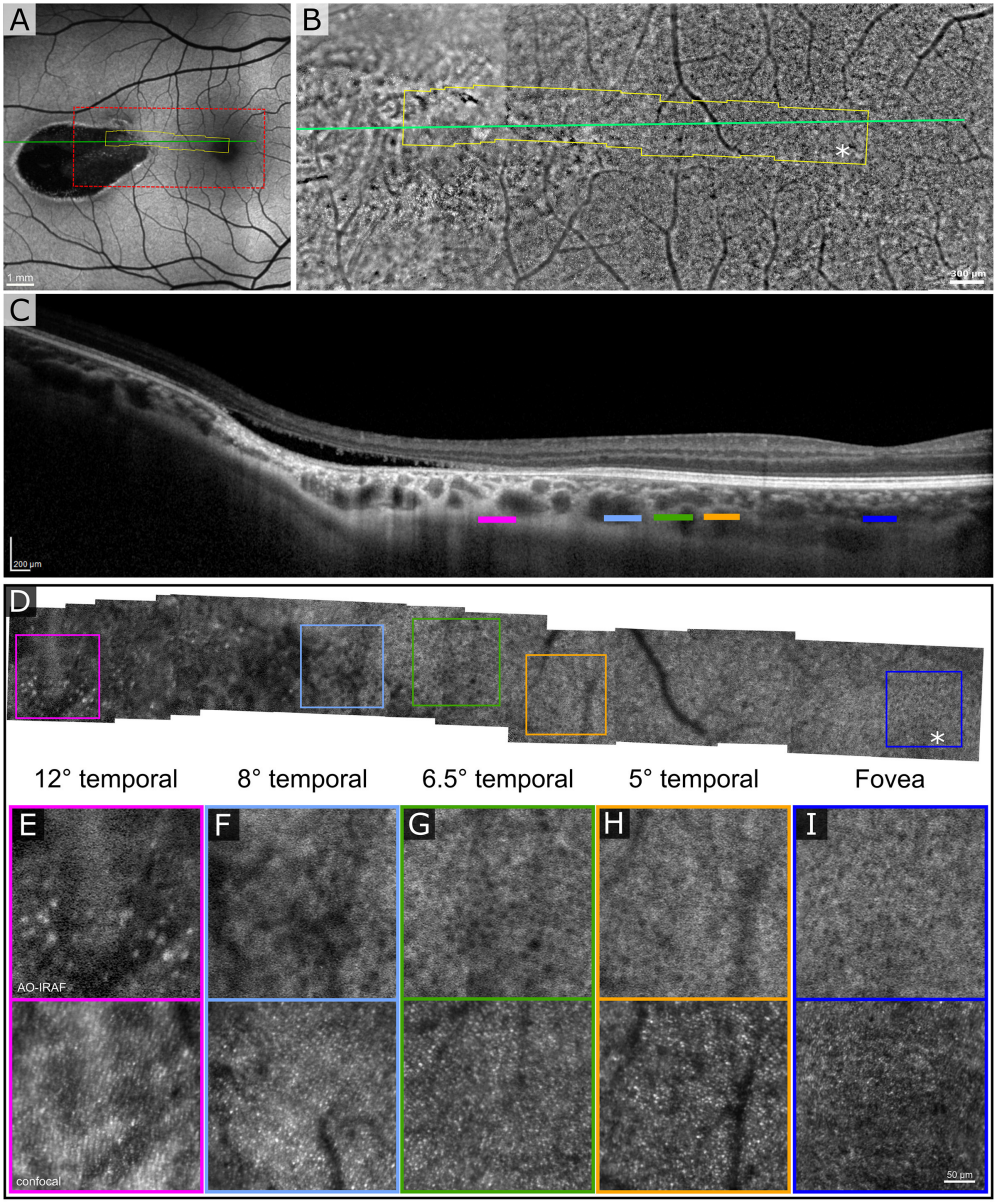
Adaptive optics (AO) assisted imaging of patient 1. **(A)** Blue autofluorescence image showing the location of the lesion with red dashed box indicating the area imaged with the flood-illumination adaptive optics (FIAO) retinal camera and green line showing the location of the OCT scan. **(B)** FIAO montage showing typical photoreceptor mosaic in the region of the fovea. Near the lesion, the cone signature becomes irregular and cannot be distinguished anymore. The yellow area indicates the area that was imaged with the AOSLO. **(C)** OCT B-scan showing the cavity, indicating Type II torpedo maculopathy. Different color bars indicate roughly the location where AO-IRAF images where obtained. **(D)** AO-IRAF montage showing typical RPE cell mosaic. The cell size increases when moving to higher eccentricities and when reaching the lesion border, the mosaic eventually disappears. **(E–I)** Zoomed images of the regions of interest from the montage. Hypo-fluorescent spots are seen in **(E)** suggesting fluorophore accumulation in the lesion itself. Bottom row showing corresponding confocal image from the areas **(E–I)**.

The first patient exhibited a typical torpedo-shaped lesion (Figures 1A–C) in the temporal macula with neurosensory detachment. Microperimetry showed decreased sensitivity in the lesion in both patients but in patient 1 with subretinal cavitation (Figure 1D), the expected zero sensitivity did not occur, suggesting some visual function remaining (Figure 1E). In the OCTA, image slices taken from approximately 150 μm below the RPE showed some dense vascularization (Figures 1F,G) and what appears to be false positive flow signal temporal to the lesion. Figure 2A shows the blue autofluorescence image with areas marked where the B-scan and flood AO imaging was done. The cone mosaic in the flood AO (Figure 2B) shows typical cone mosaic with structure disappearing with eccentricity when approaching the lesion (yellow box indicating the AO-IRAF imaging area). This is consistent with the structural information on the B-scan (Figure 2C), showing the cavity fully starting at the edge of the AO-IRAF montage (Figure 2D). AO-IRAF showed the RPE mosaic (Figures 2E–I) visible from the fovea up to the margin of the lesion with an expected increase in cell size with increasing eccentricity. At the margin of the lesion, we observed heterogeneity in cell sizes as well as a decrease in the fluorescence signal corresponding to areas larger than individual RPE cells. Within the lesion, few individual RPE cells were seen on AO-IRAF and we observed several hyper-fluorescent spots with a diameter of 15–20 μm (Figure 2E).

**Figure 3:**
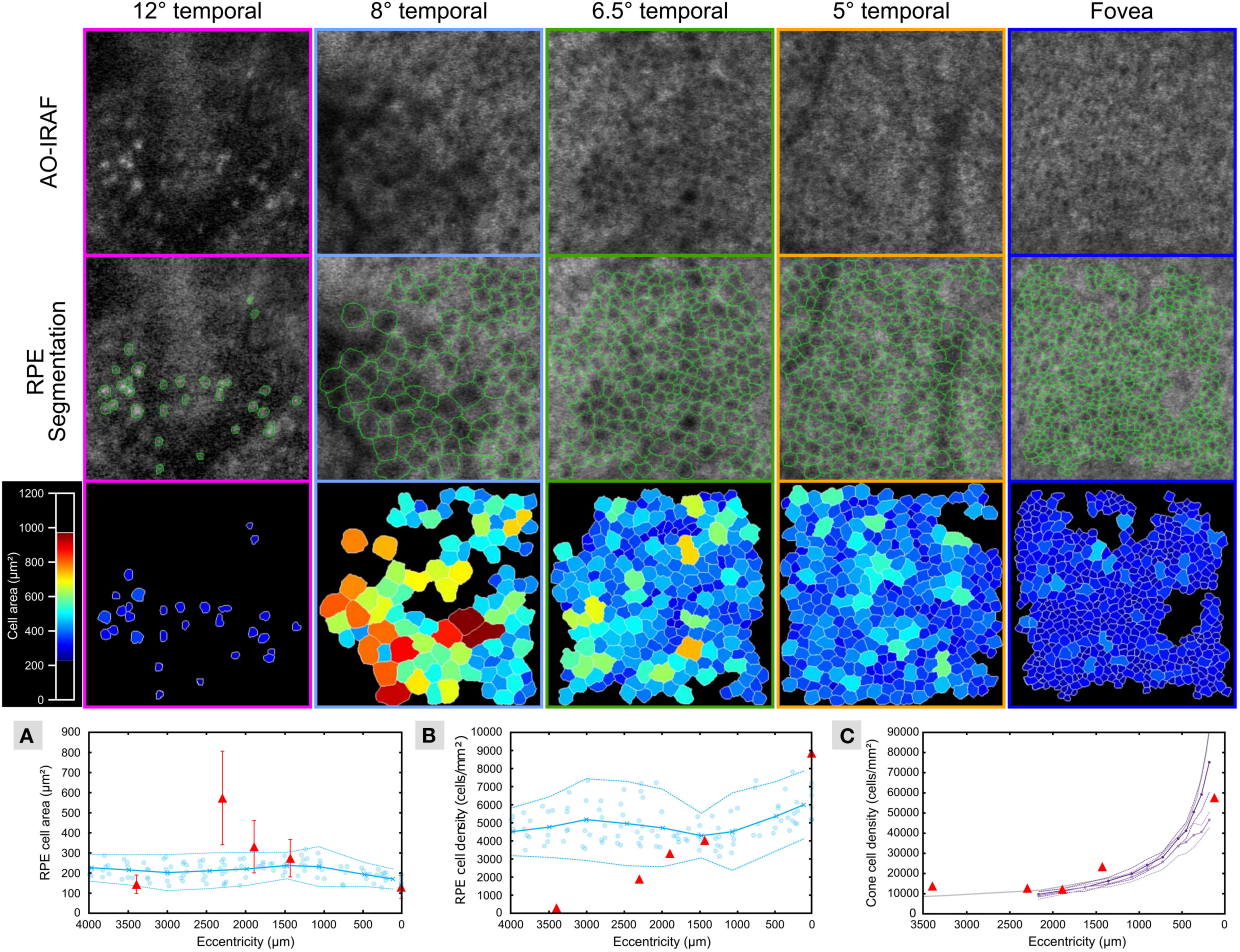
RPE cells are altered in TM outside of the lesion area. AO-IRAF images from patient 1 (top row; see Figures 2E–I) were semi-automatically segmented (middle row; green outlines). The bottom row of images shows the segmentation images colored by cell area. Near the fovea (far right images), the cells were relatively uniform in size across the region of interest, while cell size heterogeneity increased with increasing eccentricity approaching the margin of the lesion. Within the lesion (left column), some punctate hyper-AF structures were seen but they did not resemble normal RPE cells. Quantitative plots compare the RPE cell area **(A)** and density **(B)** from this patient (red triangles in A and B, respectively) to the normative data from Granger et al. (29) (blue circles are individual data points; circle markers and solid line show mean; dotted lines are ± 2 SD). Average cell area was greater than the normal range at the margin of the lesion (see data point at approximately 2,300 μm) and greater than 400 μm away from the lesion (see data point at approximately 1,900 μm), while closer to the fovea (approximately 1,400 μm) and at the fovea (0 μm) the average cell area was within the normal range. Similarly, RPE cell density began to diverge from the normal range at the 1,900 μm measurement point and fell below 2 standard deviations from normal at the lesion margin (approximately 2,300 μm) and within the lesion (approximately 3,400 μm). Cone density was measured in 100 × 100 μm FOV within each RPE ROI **(C)** and was close to the range expected for normal eyes (violet and purple lines plot the mean cone density from the *in vivo* AOSLO data of Song et al. (30) for their younger and older cohort, respectively; dashed lines are ± 2SEM; gray line is the mean cone density from the histology data of Curcio et al. (31).

Figure 3 shows the segmentation of RPE cells from the different eccentricities (from 0 to 3,400 μm) from patient 1. Average cell area (Figure 3A) was greater than the normal range [data from Granger et al. (29)] at the margin of the lesion (see data point at approximately 2,300 μm) and greater than 400 microns away from the lesion (see data point at approximately 1,900 μm), while closer to the fovea (approximately 1,400 μm); and at the fovea (0 μm), the average cell area was within the normal range. The RPE cell density (Figure 3B) deviated from the normative data at the 1,900 microns measurement point and fell below 2 standard deviations from normal at the lesion margin (approximately 2,300 μm) and within the lesion (approximately 3,400 μm). The cone density was measured in 100 × 100 μm FOV within each RPE ROI (Figure 3C) and was close to the range expected for normal eyes.

**Figure 4:**
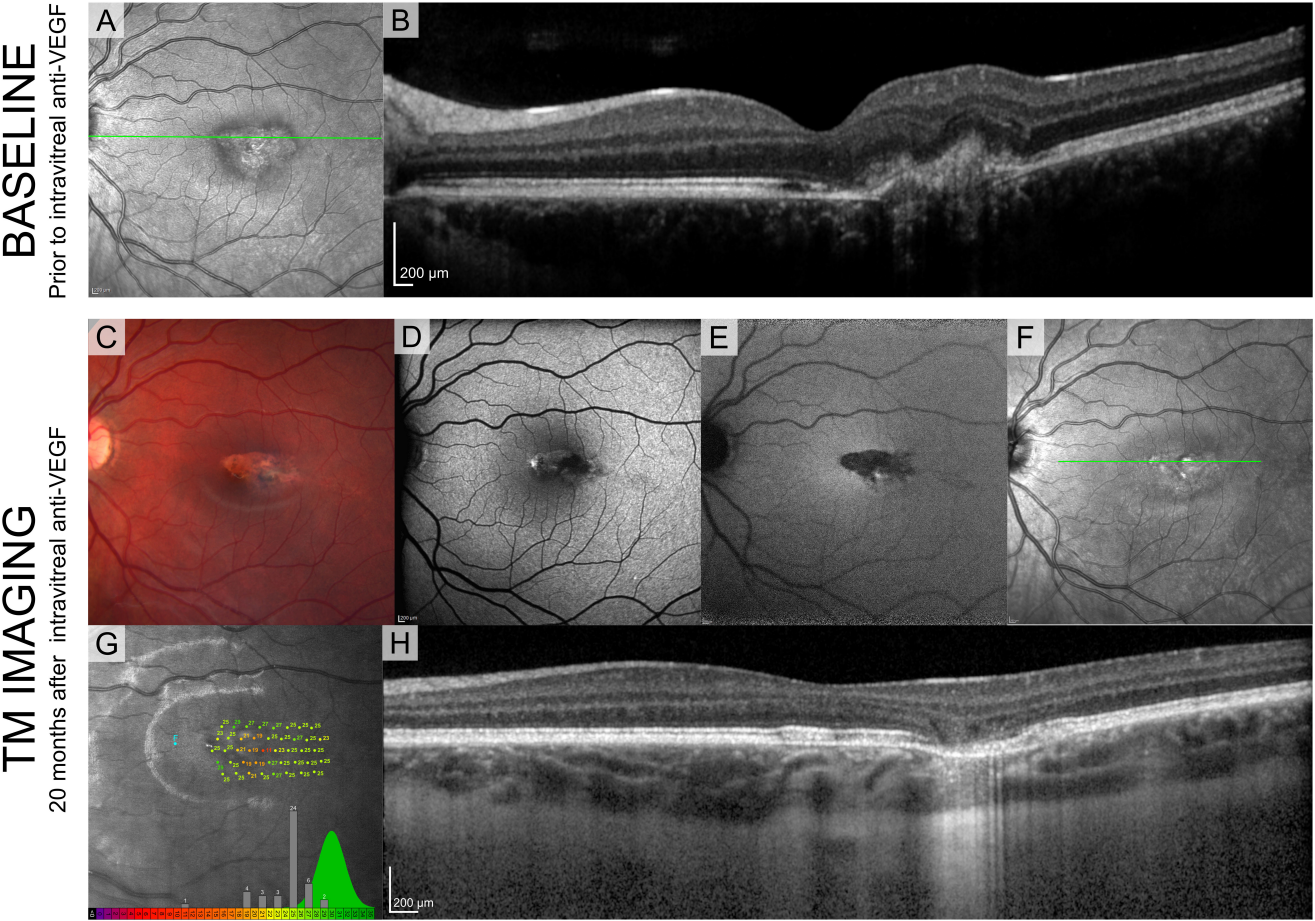
Standard clinical imaging of patient 2. **(A,B)** SLO reflectance image and OCT B-scan taken from the location marked with a green line before the injection. **(C)** Fundus photograph 20 months after the injection. **(D-F)** SLO images from Spectralis (BAF, IRAF, reflectance) showing reduced FAF signal from the lesion. **(G)** Sensitivity estimates from MAIA show decreased values but compared to patient 1 they are substantially higher, suggesting intact visual sensitivity within the lesion. Green distribution represents normative data from the manufacturer's database. **(H)** OCT B-scan taken from the location marked with a green line in **(D)**.

Since the second patient (Figure 4) was treated with intravitreal injection, we have shown the SLO fundus image, and an OCT B-scan taken prior to the injection in Figures 4A,B. A characteristic torpedo shaped lesion with irregular margins on its temporal aspect is seen in Figure 4C. Standard clinical FAF imaging shows reduced AF signal (Figures 4D-F) from the lesion area and substantial light penetration to the choroid on the OCT B-scan (Figure 4H) suggesting extensive disruption to the RPE. As in patient 1, microperimetry in patient 2 showed reduced sensitivity within the lesion but it was not reduced to same extent seen in patient 1 (Figure 4D).

**Figure 5:**
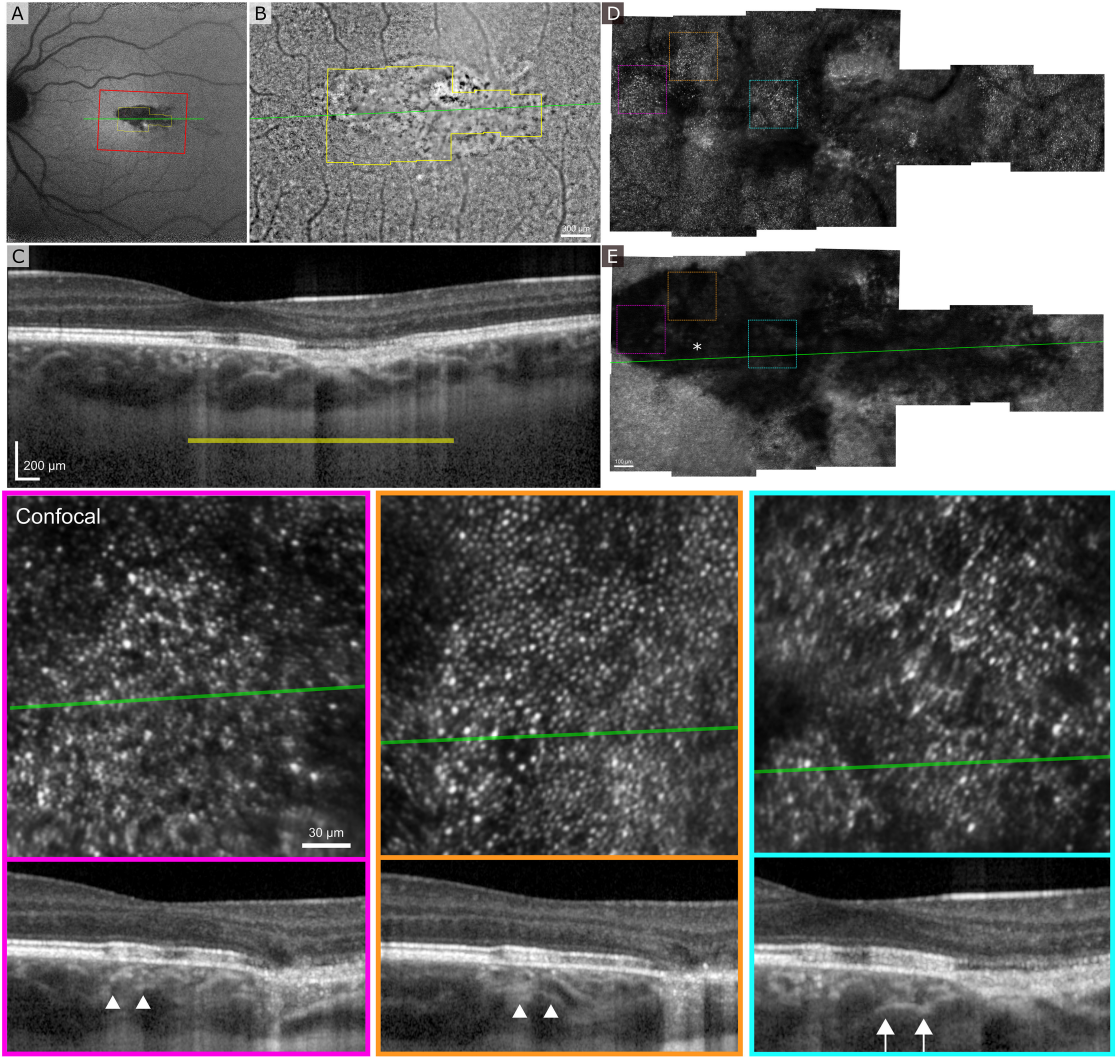
Adaptive optics ophthalmoscopy imaging of the patient 2. **(A)** A clinical SLO reflectance image showing the location of the FIAO montage with red box and OCT B-scan with a green line going through the lesion. **(B)** FIAO montage showing typical photoreceptor mosaic outside the lesion. The yellow outline indicates the area imaged using AOSLO, with yellow bar showing the FIAO image location in the OCT B-scan. **(C)** B-scan showing extensive damage at the RPE layer with slightly increased light penetration to the choroid. **(D)** Confocal AOSLO montage of the lesion area. Compared to FIAO, the cone mosaic is sharper and has more contrast due to confocality and rejection of out-of-focus light. Even within the lesion patches of photoreceptor mosaic are seen. **(E)** AO-IRAF shows highly reduced FAF from the lesion throughout, even within areas where the photoreceptor mosaic seemed healthy, suggesting that the RPE underlying the photoreceptors is damaged. The colored dashed squares mark the location of the zoomed in areas at the bottom with colors indicating the areas. The B-scan locations are marked with a green line and in the B-scans the white arrow heads show the edges of the zoomed in area. The location of the fovea is marked with an asterisk in **(E)**.

Figure 5A shows the clinical SLO image with red rectangle indicating the area imaged with flood-illumination AO (Figure 5B) and green line showing the location of the B-scan (Figure 5C). Despite a very low AO-IRAF signal in some areas (Figure 5E), the cone mosaic was well defined and clearly visible at many locations within the lesion on FIAO (Figure 5B) and confocal AOSLO (Figure 5D). AO-IRAF did now show the typical RPE mosaic within the lesion but trace signatures of the mosaic and RPE cells are seen in the bottom left corner of the montage just outside the margins of the TM lesion as well as at the right edge of the AO-IRAF montage.

## Discussion

Herein, we show the *in vivo* morphology of both the cone and RPE cell mosaics in TM for the first time. These findings suggest that outside the TM lesion, RPE morphometry is relatively normal. However, near the margin of the lesion, we see marked changes in RPE morphometry characteristic of a disrupted cell mosaic. Within the TM lesion, we observed differences between the two cases that may reflect differences between type I and type II lesions. In patient 1 (Figures 1–3), with a type II lesion (neurosensory detachment), we saw reduced AO-IRAF compared to the normal appearing areas outside the lesion but there was still well-defined structure within the lesion such as several areas of hyper-autofluorescence similar in size to individual RPE cells. It is possible that these hyper-autofluorescent areas represent RPE cells that have been altered due to the TM and accumulated additional NIR fluorophores. Another possible explanation of the hyperautofluorescence in the lesion borders could be RPE rounding/stacking but we did not see evidence of this when we examined the B-scan stack going through the lesion, particularly the hyperAF areas.

In the second case, there was very little well-defined RPE structure within the TM lesion; though some areas of hyper- and hypo-autofluorescence were seen, no hyper-AF structures were seen that had a similar appearance to what was seen in the first case. Since the photoreceptor mosaic in the confocal channel was well-resolved we do not think that this is an artifact due to poor image quality but rather is reflective of the distribution of fluorophores within the RPE.

Compared to other studies that have implemented commercial flood-illumination adaptive optics retinal cameras in their studies, the confocal reflectance images show similar morphology with no clear photoreceptor mosaic visible (21, 22). Most likely the photoreceptors are there but are misaligned resulting in an altered reflectance signal.

Interestingly, some retinal sensitivity was preserved within the lesion bounds in both patients. This was unsurprising in patient 2, with the type I TM lesion, since it appeared both clinically and on AO-IRAF that sufficient RPE was present to support some photoreceptor survival and function. It was more surprising to see some evidence of photoreceptor function in patient 1, in whom the RPE appeared macroscopically and on AO-IRAF to be mostly obliterated. Given that eyes with complete and chronic RPE atrophy often exhibit severe outer retinal disruption, this observation led us to speculate that neurosensory detachment from a pathological RPE might have been relatively protective to the photoreceptors. It also caused us to question whether TM is congenital, for if so, the observations suggest that it must arise relatively late in development, after outer retinal development and photoreceptor packing are completed. Considering our sensitivity losses in comparison to the literature, it appears that sensitivity losses in TM vary across a spectrum ranging from little to mild losses, such as the case of patient 3 with Wong et al. (9) to moderate losses such as seen in our case 2 to the more severe losses accompanied by cavitation such as in our case 1 and Wong's patient 4.

As patient 2 underwent a single intravitreal bevacizumab injection prior to being imaged in our study, it is possible that that previous neovascularization might have altered the structure of the RPE. However, based on the literature (3, 14), it appears relatively common to have neovascularization on this rare condition, especially with type I. It is possible that there are some different alterations to the retina in TM with and without neovascularization. Without additional data, it is hard to interpret the level of influence on the AF signal itself.

Although the sample size for this descriptive study was limited by the low prevalence of TM, we were able to image both variants, type I and II, in detail. Clinically, TM can present with varying appearance and new possible lesion subtypes (32) may still be discovered such as those presenting with vitelliform material (33). Near infrared autofluorescence imaging using adaptive optics is an exciting new field and more quantitative results are possible in future studies.

## Conclusion

Contrary to our expectation that the transition from abnormal to normal cellular mosaics would be relatively abrupt, with abnormalities confined to the immediate vicinity of the lesion, we found that RPE morphometric alterations extended well beyond the bounds of the clinically defined TM lesion. Additionally, the demarcation between abnormal and normal cone and RPE mosaics were not aligned, as cone density appeared to fall within the normal range at each of the locations where RPE cells were quantified in case 1, even though RPE cell morphometry was abnormal in several of these locations. The fluorescence signal that we detect with adaptive optics ophthalmoscopy can show alterations in the RPE when the structural cone images still appear normal. This was shown here in the case of TM patients, but this methodology can be adapted to various diseases and when accompanied by complimentary imaging modalities such as OCT, is a promising tool for clinicians to study the health of the retina.

## Data Availability Statement

The raw data supporting the conclusions of this article will be made available by the authors, without undue reservation.

## Ethics Statement

The studies involving human participants were reviewed and approved by University of Pittsburgh Institutional Review Board. The patients/participants provided their written informed consent to participate in this study.

## Author Contributions

ER and KV conceived and conducted the experiment(s) and also drafted the manuscript. ER, KD, and KV analyzed and interpreted the results. VS conducted the clinical imaging. JM and AE critically revised the manuscript for important intellectual content. All co-authors carefully reviewed the final manuscript.

## Funding

This research was supported by departmental startup funds from the University of Pittsburgh to ER. This work was also supported by the NIH CORE Grant P30 EY08098 to the University of Pittsburgh, Department of Ophthalmology, the Eye and Ear Foundation of Pittsburgh, NVIDIA GPU Grant Program and from an unrestricted grant from Research to Prevent Blindness, New York, NY, USA.

## Conflict of Interest

Some aspects of this work include technologies that ER is an inventor on for patents that are owned by the University of Rochester (US Patent No.: US 10,123,697 and US 10,092,181). The remaining authors declare that the research was conducted in the absence of any commercial or financial relationships that could be construed as a potential conflict of interest.

## Publisher's Note

All claims expressed in this article are solely those of the authors and do not necessarily represent those of their affiliated organizations, or those of the publisher, the editors and the reviewers. Any product that may be evaluated in this article, or claim that may be made by its manufacturer, is not guaranteed or endorsed by the publisher.
