# Supplementary figures and images for: Multimodal Imaging of Torpedo Maculopathy With Fluorescence Adaptive Optics Imaging of Individual Retinal Pigmented Epithelial Cells
Source: Front Med (Lausanne). 2021 Dec 9;8:769308. doi: 10.3389/fmed.2021.769308 (PMC8698897; doi:10.3389/fmed.2021.769308)

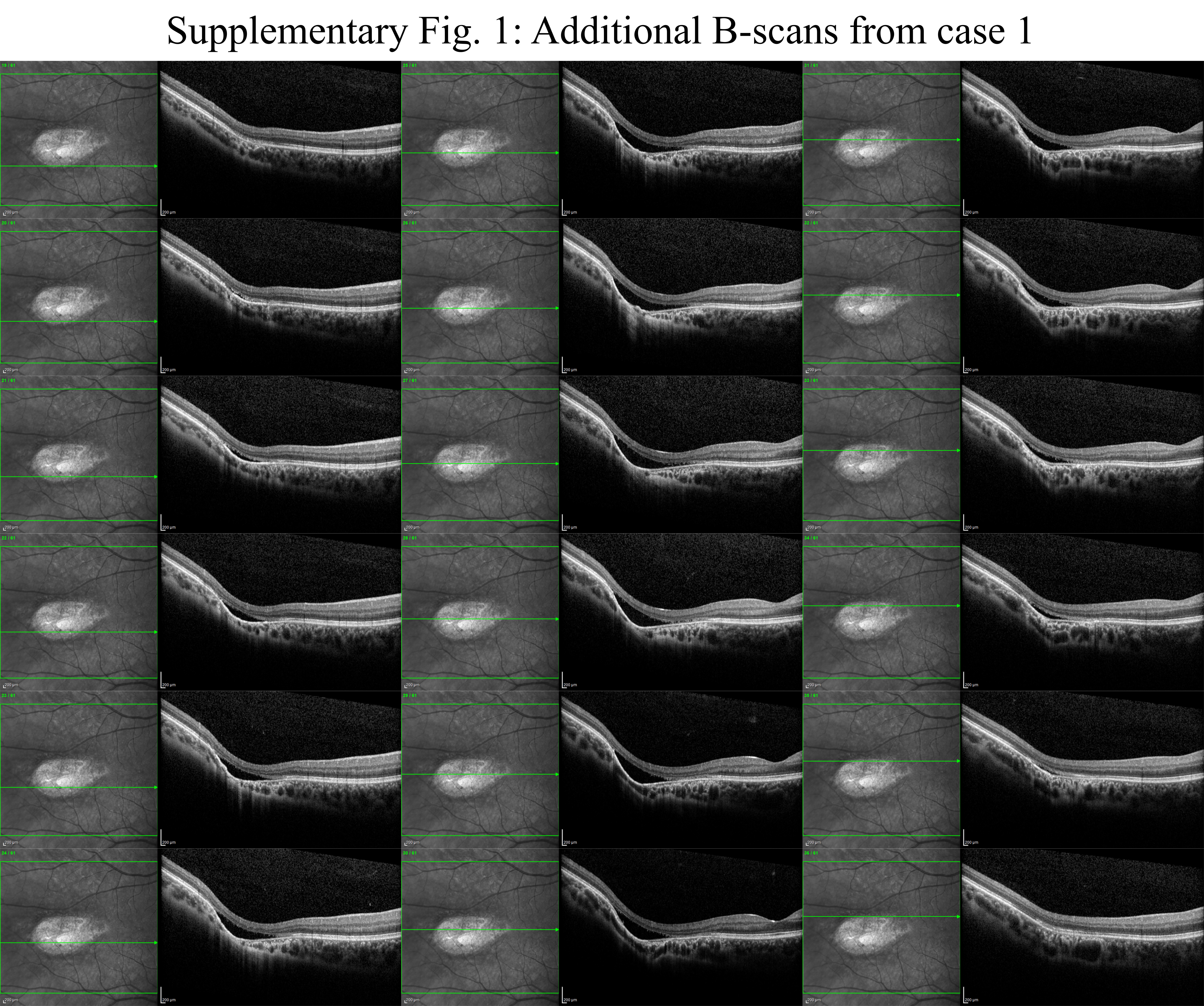

Supplement: Supplementary file 1 [file Image_1.JPEG]

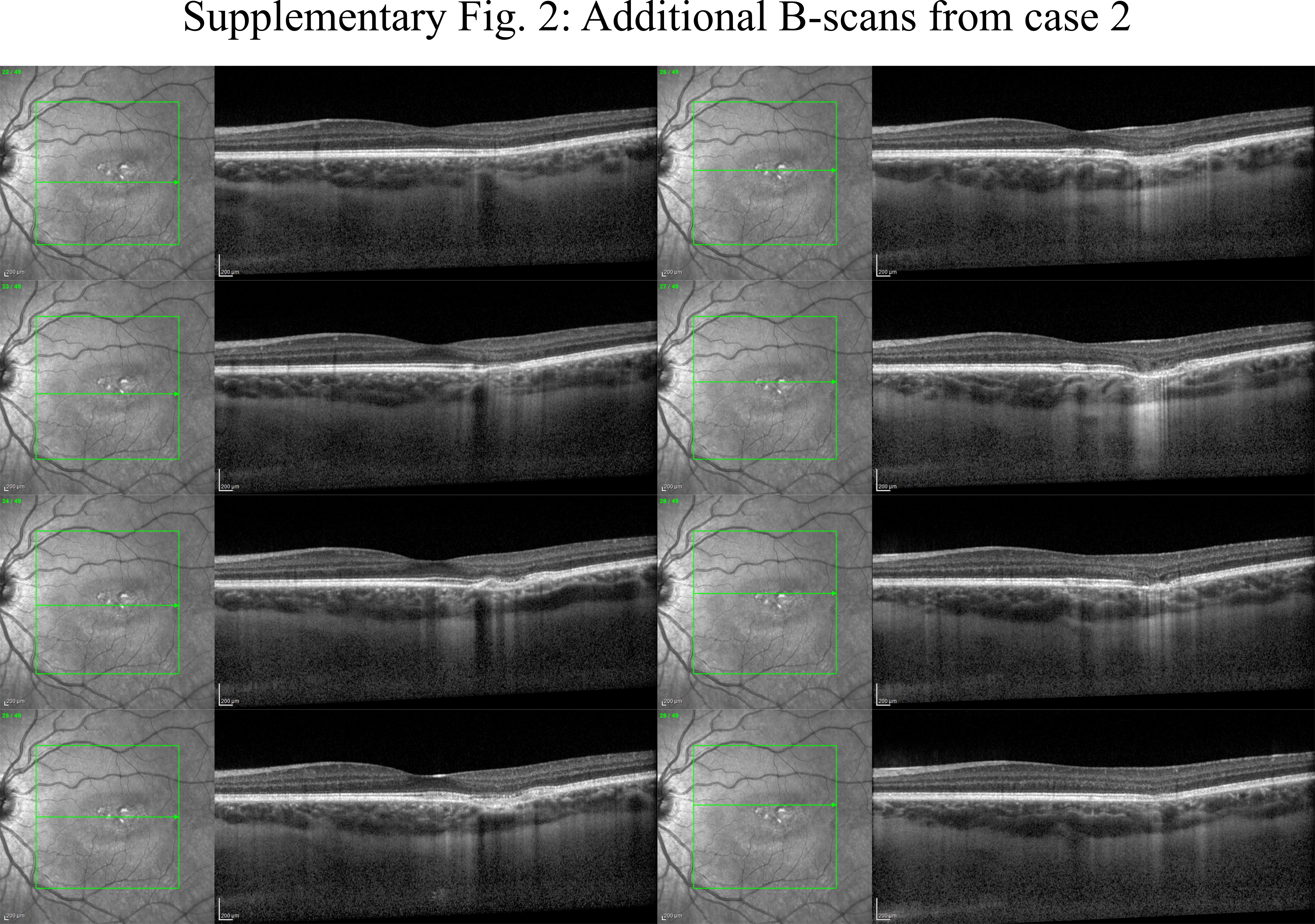

Supplement: Supplementary file 2 [file Image_2.JPEG]
